# Supplementary material for: Dynalign II: common secondary structure prediction for RNA homologs with domain insertions
Source: Nucleic Acids Res. 2014 Nov 21;42(22):13939–48. doi: 10.1093/nar/gku1172 (PMC4267632; doi:10.1093/nar/gku1172)
Supplement: SUPPLEMENTARY DATA [file supp_gku1172_nar-02021-z-2014-File012.zip › manual/javadoc/overview-summary.html]

Overview


JavaScript is disabled on your browser.


- Overview
- Package
- Class
- Tree
- Index
- Help

- Prev
- Next

- Frames
- No Frames

- All Classes

These are the Javadocs for all Java folders in the RNAstructure software package.

See: Description

Packages

| Package | Description |
| --- | --- |
|  |  |
| --- | --- |
| RNAstructure\_java\_drawing.source | A package that encapsulates all the Java classes that handle structure and dot plot drawing. |
| RNAstructure\_java\_drawing.source.dialogs | A package that holds all Java drawing dialogs. |
| RNAstructure\_java\_drawing.source.menus | A package that holds all menus used in the drawing framework. |
| RNAstructure\_java\_drawing.source.proxy | A package that holds all Java drawing classes generated by SWIG that connect the C++ back end to the Java front end. |
| RNAstructure\_java\_drawing.source.runners | A package that holds the run templates that are used when the Java drawing framework is wrapped into self-contained executables. |
| RNAstructure\_java\_interface.source | A package that encapsulates all the Java classes that comprise the RNAstructure Java interface. |
| RNAstructure\_java\_interface.source.menus | A package that holds all menus used in the Java RNAstructure interface. |
| RNAstructure\_java\_interface.source.startup | A package that holds all Java RNAstructure interface classes that are directly called at the Java interface startup. |
| RNAstructure\_java\_interface.source.windows | A package that holds all internal windows used in the drawing framework. |
| RNAstructure\_java\_utilities | A package that holds a variety of utilities classes. |

These are the Javadocs for all Java folders in the RNAstructure software package.
  
These folders encompass the Java drawing framework, the Java RNAstructure interface, and the Java utilities classes.
  
  
Some classes and packages are backed by C++ files; these C++ files are documented with Doxygen documentation.

- Overview
- Package
- Class
- Tree
- Index
- Help

- Prev
- Next

- Frames
- No Frames

- All Classes
